# Validity of inertial sensor based 3D joint kinematics of static and dynamic sport and physiotherapy specific movements

**DOI:** 10.1371/journal.pone.0213064

**Published:** 2019-02-28

**Authors:** Wolfgang Teufl, Markus Miezal, Bertram Taetz, Michael Fröhlich, Gabriele Bleser

**Affiliations:** 1 Department of Computer Science, Technische Universität Kaiserslautern, Kaiserslautern, Germany; 2 Department of Sport Science, Technische Universität Kaiserslautern, Kaiserslautern, Germany; University of Memphis, UNITED STATES

## Abstract

3D joint kinematics can provide important information about the quality of movements. Optical motion capture systems (OMC) are considered the gold standard in motion analysis. However, in recent years, inertial measurement units (IMU) have become a promising alternative. The aim of this study was to validate IMU-based 3D joint kinematics of the lower extremities during different movements. Twenty-eight healthy subjects participated in this study. They performed bilateral squats (SQ), single-leg squats (SLS) and countermovement jumps (CMJ). The IMU kinematics was calculated using a recently-described sensor-fusion algorithm. A marker based OMC system served as a reference. Only the technical error based on algorithm performance was considered, incorporating OMC data for the calibration, initialization, and a biomechanical model. To evaluate the validity of IMU-based 3D joint kinematics, root mean squared error (RMSE), range of motion error (ROME), Bland-Altman (BA) analysis as well as the coefficient of multiple correlation (CMC) were calculated. The evaluation was twofold. First, the IMU data was compared to OMC data based on marker clusters; and, second based on skin markers attached to anatomical landmarks. The first evaluation revealed means for RMSE and ROME for all joints and tasks below 3°. The more dynamic task, CMJ, revealed error measures approximately 1° higher than the remaining tasks. Mean CMC values ranged from 0.77 to 1 over all joint angles and all tasks. The second evaluation showed an increase in the RMSE of 2.28°– 2.58° on average for all joints and tasks. Hip flexion revealed the highest average RMSE in all tasks (4.87°– 8.27°). The present study revealed a valid IMU-based approach for the measurement of 3D joint kinematics in functional movements of varying demands. The high validity of the results encourages further development and the extension of the present approach into clinical settings.

## 1 Introduction

The assessment of functional movements has become an important part of physical therapy and the practice of sports medicine [1]. Functional movement, or fundamental movement, describes a kind of complex 3D movement along several jointsand the incorporating muscle synergies. Three functional movements used for analysis in these professions are the bilateral squat (SQ) [2], the single-leg squat (SLS) [3,4], and the countermovement jump (CMJ) [5,6]. These functional movements can provide information about rehabilitation status or injury risks [7,8]. It is not only important to assess the movement performance (e.g., speed, repetitions, jump height, etc.), but also how the movement is executed. The joint kinematics recorded during functional movements can reveal information about the correctness of the movement. Optical motion capture systems (OMC) are commonly used to evaluate joint kinematics [9]. However, OMC systems do not always allow the subject to move in his/her accustomed environment. In this context, inertial measurement units (IMU) present a local independent alternative [10], and their use has been widely accepted. A single IMU has been used to examine the horizontal and vertical displacement of the pelvis during a CMJ [11], to evaluate kinetic data from three different jumping tasks [12], and to assess the performance of a CMJ [13]. The 3D joint kinematics of the hip and ankle, as well as the 1D joint kinematics of the knee during a SLS, were evaluated using a set of three IMU’s attached to the pelvis, thigh and shank [7]. However, some of these studies only used one IMU and focused only on performance measures [11–13]. Others examined joint kinematics but did not compare their results to a reference system [7].

The accuracy of IMU-based joint kinematics, or spatio-temporal parameters, have been examined in several studies on gait analysis [14–18]. Despite the increasing application of IMU’s the availability of validity studies regarding the measurement of 3D joint kinematics in functional movements is limited. Al-Amri et al. [14] examined the reliability and validity of SQ and vertical jumping 3D joint kinematics of the lower extremity. However, they failed to report detailed results concerning the validity of the transversal and frontal plane joint kinematics. Robert-Lachaine et al. [19] evaluated the accuracy of full body joint kinematics during ergonomic tasks. However, IMU’s aim to be used in rehabilitation and sports medicine. The functional movements mentioned above differ from common gait inasmuch as they incorporate almost no global translation, usually demand higher ranges of motion (ROM) and, in the case of the CMJ, higher global accelerations. Thus, the validity of IMU-based 3D joint kinematics of slow and dynamic functional movements has to be further investigated.

A recently described sensor-fusion algorithm for the estimation of 3D IMU kinematics [20,21] revealed long-term stable results of the joint kinematics estimation of the lower extremities in a gait analysis [18]. These results proved to be unaffected by drift, despite omitting magnetometer information. Based on those results, the primary aim of this study was to evaluate the performance of this algorithm in estimating 3D joint kinematics in dynamic, clinically-relevant movements with high ROM. As in [18], this evaluation focuses on the technical differences between IMU- and OMC-based joint kinematics associated to algorithmic issues. Thus, the IMU initialization, calibration, and the biomechanical model were derived from the OMC system.

Like optical markers, IMU’s are prone to artefacts caused by the displacement of the sensor and that of the underlying tissue with respect to the bone [22]. These artefacts are commonly referred to as soft tissue artefacts (STA). The effect of STA on OMC- and IMU-based joint kinematics [9,23–25] and possible compensation mechanisms [26–29] have been intensively examined in the recent literature. It was an objective of the present study to minimize the differences between the two systems associated to STA. Researchers use rigid marker clusters (RMC) affixed to the IMU for the calculation of OMC-based joint kinematics to achieve this goal [11,17,19]. The alternative calculation of OMC-based joint kinematics involves skin markers attached to anatomical landmarks. Examinations have shown that the OMC-based joint kinematics derived from RMC are less susceptible to errors caused by STA than markers mounted on bony prominences [30]. However, both methods are commonly used in research as well as clinical settings. Furthermore, few studies have reported results that compare IMU joint kinematics with OMC joint kinematics based on skin markers attached to anatomical landmarks [14,16,31].

Therefore, the second aim of the study was to compare IMU-based joint kinematics with OMC- based joint kinematics derived from skin markers attached to anatomical landmarks instead of RMC.

## 2 Methods and materials

### 2.1 Subjects and data acquisition

Twenty-eight healthy subjects (15 females, 13 males; 24 ± 2.70 years; 70 ± 12.70 kg; 176 ± 9.00 cm) participated in the study. The subjects were recruited via e-mail, bulletins, and presentations in specific lectures at the local university. The study was approved by the local ethical committee of the Technische Universität Kaiserslautern (TUK) and meets the criteria of the declaration of Helsinki. After receiving all relevant study information, the participants signed an informed consent to the study including a permission to publish the data. A test session consisted of one static neutral zero position (n-pose) sequence [32]; three trials of five, right-legged SLS; three trials of five SQ; and, three trials of three CMJ. A SLS and SQ cycle was defined as the time from maximum knee extension to the next maximum knee extension. A CMJ cycle was defined as the time from the first downward movement of the pelvis marker until it reached the next static phase. Every SLS, SQ, and CMJ considered for evaluation was normalized to 100% movement cycle.

OMC lower extremity 3D joint kinematics was captured using OptiTrack Motive 1.10 (NaturalPoint, Inc., Oregon, USA). IMU raw data was recorded by means of seven Xsens MTw Awinda IMU and Xsens MVN Biomech software version 4.3.7 (Xsens Technologies BV, Enschede, Netherlands). Both systems were synchronized and recorded at 60 Hz.

All IMU’s were activated 20 minutes prior to each test session and before the subjects were instrumented. Before testing, a static trial was conducted by laying all IMU’s on the ground for about 10 seconds. These measurements were used to estimate and subtract the gyroscope bias according to [33].

**Fig 1:**
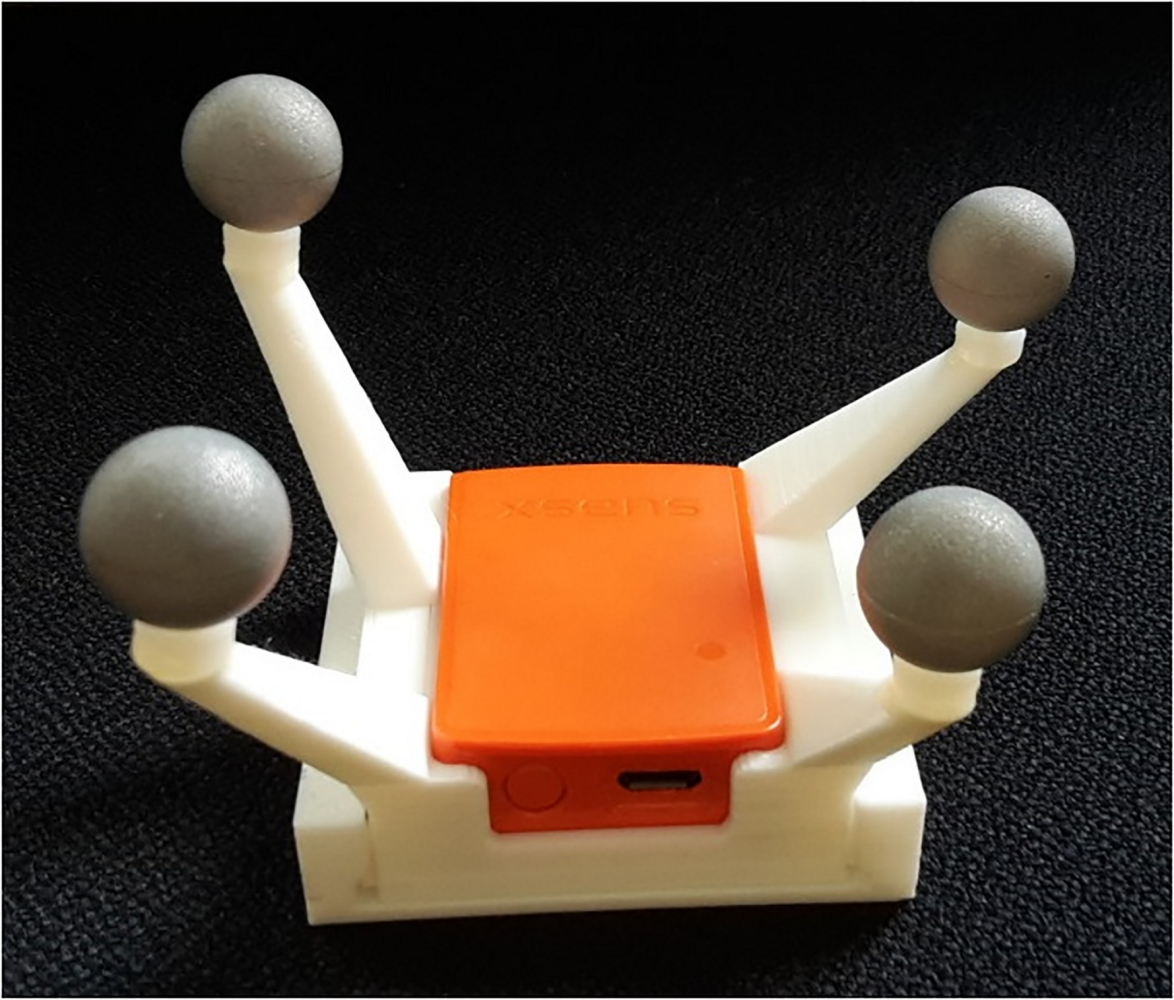
Sensor fixation. Inertial Measurement Unit (IMU) inserted into a matched, 3D-printed Rigid Marker Cluster (RMC).

**Fig 2:**
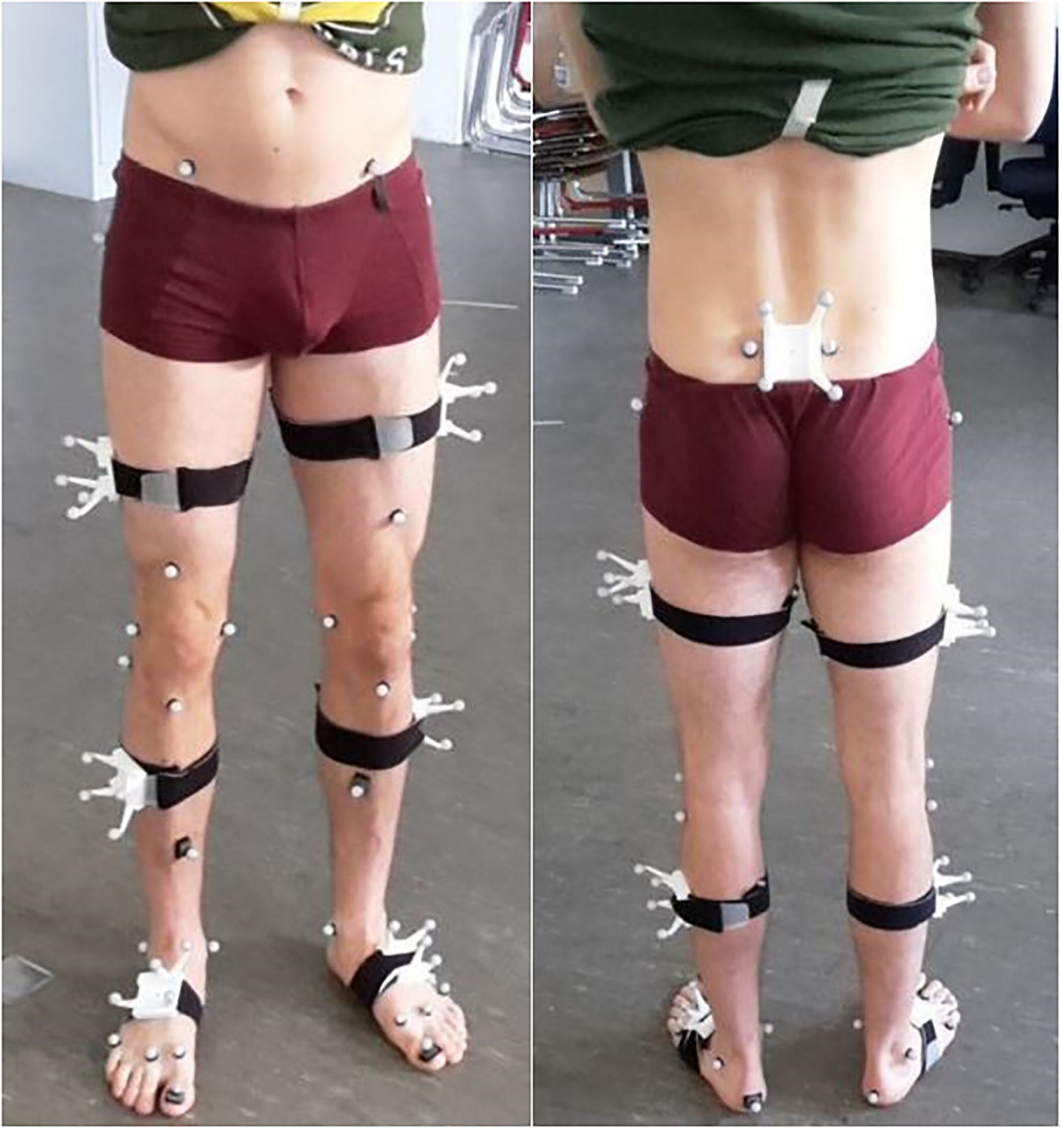
Marker protocol and IMU placement. Retroreflective markers attached to the anatomical landmarks and the RMC. IMU are not inserted in the RMC in this schematic picture.

Thirty-two retroreflective markers were attached to anatomical landmarks according to [34]. Six additional markers were applied following the OptiTrack recommendations. Each IMU was inserted into a matching 3D printed box equipped with four retroreflective markers (Fig 1). These boxes were attached to the body segments of the lower extremities using straps and double-sided adhesive tape. The IMU and RMC for the thigh and shank were placed according to recommendations by Manal et al. [35,36]. The IMU and RMC attached to the pelvic segment was placed on top of the sacrum according to Cutti et al. [37]. The IMU and RMC for the feet could only be placed on the dorsum of the foot due to their size and visibility. Schematic marker protocol and IMU placement are shown in Fig 2.

To exclude possible errors in the IMU-derived data based on different coordinate systems, calibration, or the initialization process, the biomechanical model [34] and IMU-to-segment calibrations were calculated based on the OMC data of the recorded n-pose sequence. Additionally, the initialization of the IMU-based kinematics estimation incorporated data from the OMC system.

The IMU raw data was processed with a sensor-fusion algorithm using an iterated extended Kalman Filter (EKF) approach based on [20] and enhanced with global translation estimation [21] that used only accelerometer and gyroscope data; magnetometer information was omitted. The complete algorithm is described in [18]. The same filter settings and tuning parameters that are described in [18] were used in this study. The estimated segment orientations were used to derive relative joint orientations. These were decomposed into joint angles using Euler angle decomposition [38]. The OMC joint angle data was calculated based on the RMC and the skin markers according to the recommendations by Visual 3D (C-Motion, Inc, Germantown, MD, USA). A more detailed description of the calibration, initialization and joint angle calculation can be found at dx.doi.org/10.17504/protocols.io.vwye7fw.

### 2.2 Statistical analysis

The analysis of the IMU system was twofold. First, the joint kinematics data from the IMU was compared to the OMC joint kinematics data based on the RMC, in order to minimize the error between the systems caused by different positioning of the IMU and markers respectively (RMC evaluation). In a secondary analysis, the IMU data was compared to the OMC data from the skin markers attached to anatomical landmarks (skin marker evaluation).

To determine the validity of the IMU system, the following statistical measures were calculated for each evaluation of the hip, knee, ankle joint, and global 3D pelvis orientations per movement cycle: the root mean squared error (RMSE), the range of motion error (ROME), as well as 95% confidence interval (CI). A Bland-Altman (BA) analysis was conducted to evaluate the bias and limits of agreement between the OMC- and IMU-based joint kinematics according to [39]. For the BA analysis, the average of all movement cycles for each subject was used. This approach was deemed appropriate given that the mean of a similar number of movement cycles is usually examined in clinical measurements [40]. Further, the coefficient of multiple correlation (CMC) was calculated for each parameter per movement cycle according to [41]. CMC values were rated according to [42]. For further interpretation, the mean of the statistical measures of all subjects and movement cycles was calculated.

An ANOVA was conducted to identify significant differences in the RMSE between the three different functional movements. Additionally, the data from a previous study [18] that used the same approach for the calculation of IMU-based 3D joint kinematics during gait, was included in the comparison. The significance level was set to α = 0.05. The Chi-square goodness-of-fit test was conducted to check for normal distribution in the data. A post hoc analysis revealed which groups differed in the case of significant p-values.

The changes in RMSE and ROME between the RMC evaluation and the skin marker evaluation were graphically represented.

The segmentation of the joint angles and all statistics were conducted in Matlab 2017 (Mathworks Inc.) using custom written scripts.

## 3 Results

### 3.1 RMC evaluation

RMSE and ROME of all joint angles for SQ, SLS and CMJ are shown in Table 1. For the readers convenience, Table 1 includes the RMSE of the gait data from [18]. S1–S3 Figs in the supporting information show the mean joint angle waveforms of one exemplary subject for all three tasks and all three planes.

**Table 1:** Results of the RMC evaluation.

	RMSE [deg] ± SD (95% CI)				ROME [deg] ± SD (95% CI)		
	Gait [18]	SQ	SLS	CMJ	SQ	SLS	CMJ
LT Hip − Abduction	1.05 ± 0.42 (0.78−1.11)	1.70 ± 0.89 (1.12−1.81)	x	1.23 ± 0.43 (1.03−1.37)	1.72 ± 1.26 (0.83−1.80)	x	1.20 ± 0.57 (0.89−1.33)
LT Hip − Rotation	1.94 ± 0.92 (1.49−2.20)	2.28 ± 1.25 (1.65−2.62)	x	1.78 ± 0.78 (1.12−1.73)	1.19 ± 0.90 (0.50−1.20)	x	1.01 ± 0.38 (0.78−1.08)
LT Hip − Flexion	1.02 ± 0.35 (0.79−1.06)	1.17 ± 0.45 (0.94−1.29)	x	1.55 ± 0.31 (1.42−1.66)	0.89 ± 0.54 (0.55−0.97)	x	1.42 ± 0.81 (0.97−1.59)
LT Knee − Abduction	1.59 ± 0.48 (1.22−1.59)	2.06 ± 0.72 (1.62−2.18)	x	2.02 ± 0.68 (1.67−2.20)	1.49 ± 0.84 (0.84−1.50)	x	2.20 ± 1.13 (1.49−2.37)
LT Knee − Rotation	2.34 ± 1.08 (1.63−2.48)	2.66 ± 1.55 (1.71−2.91)	x	2.75 ± 0.97 (2.39−3.14)	1.59 ± 0.87 (0.98−1.66)	x	1.85 ± 0.86 (1.41−2.07)
LT Knee − Flexion	1.47 ± 0.34 (1.25−1.51)	1.10 ± 0.28 (0.93−1.15)	x	1.83 ± 0.46 (1.54−1.89)	1.29 ± 0.62 (1.03−1.51)	x	1.72 ± 1.00 (1.10−1.87)
LT Ankle − Inversion	1.61 ± 0.39 (1.42−1.73)	1.83 ± 0.74 (1.31−1.88)	x	2.46 ± 0.73 (1.98−2.55)	2.06 ± 1.30 (1.29−2.30)	x	2.43 ± 1.17 (1.82−2.73)
LT Ankle − Rotation	2.16 ± 0.68 (1.80−2.33)	2.01 ± 1.15 (1.50−2.39)	x	2.92 ± 0.94 (2.50−3.22)	0.91 ± 0.62 (0.51−0.99)	x	2.28 ± 0.83 (1.68−2.32)
LT Ankle − Flexion	1.55 ± 0.34 (1.46−1.72)	1.22 ± 0.51 (0.92−1.32)	x	2.48 ± 0.53 (2.29−2.71)	0.60 ± 0.23 (0.47−0.65)	x	2.11 ± 0.97 (1.60−2.35)
RT Hip − Abduction	1.09 ± 0.54 (0.63−1.05)	1.39 ± 0.80 (0.78−1.40)	1.26 ± 0.68 (0.78−1.30)	1.30 ± 0.61 (0.90−1.37)	1.29 ± 0.78 (0.74−1.34)	1.34 ± 0.87 (0.77−1.45)	1.14 ± 0.61 (0.73−1.21)
RT Hip − Rotation	1.64 ± 1.00 (1.00−1.77)	1.77 ± 1.05 (1.21−2.02)	2.11 ± 0.99 (1.51−2.28)	2.01 ± 1.07 (1.29−2.12)	1.03 ± 0.83 (0.38−1.02)	0.69 ± 0.38 (0.39−0.69)	1.05 ± 0.63 (0.79−1.28)
RT Hip − Flexion	0.98 ± 0.51 (0.68−1.07)	1.07 ± 0.30 (0.96−1.19)	1.01 ± 0.69 (0.60−1.13)	1.44 ± 0.35 (1.23−1.51)	0.82 ± 0.49 (0.51−0.88)	0.95 ± 0.84 (0.46−1.11)	1.32 ± 0.72 (0.95−1.51)
RT Knee − Abduction	1.26 ± 0.51 (0.90−1.30)	1.54 ± 0.75 (0.96−1.54)	1.49 ± 0.74 (1.18−1.76)	1.48 ± 0.51 (1.22−1.62)	1.40 ± 0.98 (0.68−1.44)	1.23 ± 0.96 (0.48−1.22)	1.33 ± 0.59 (0.97−1.43)
RT Knee − Rotation	1.75 ± 0.63 (1.38−1.87)	1.86 ± 1.28 (0.80−1.79)	2.06 ± 1.46 (1.30−2.44)	2.29 ± 1.14 (1.53−2.41)	0.94 ± 0.82 (0.36−0.99)	0.74 ± 0.36 (0.45−0.74)	1.46 ± 0.82 (0.97−1.60)
RT Knee − Flexion	1.51 ± 0.43 (1.31−1.64)	1.01 ± 0.27 (0.84−1.05)	1.03 ± 0.63 (0.62−1.10)	1.83 ± 0.38 (1.61−1.91)	1.13 ± 0.48 (0.99−1.37)	0.88 ± 0.54 (0.51−0.93)	1.80 ± 0.92 (1.02−1.73)
RT Ankle − Inversion	1.33 ± 0.35 (1.09−1.36)	1.18 ± 0.51 (0.85−1.24)	1.26 ± 0.71 (0.80−1.35)	1.77 ± 0.48 (1.42−1.79)	0.64 ± 0.37 (0.43−0.71)	0.77 ± 0.32 (0.63−0.88)	1.71 ± 0.65 (1.50−2.00)
RT Ankle − Rotation	1.52 ± 0.41 (1.27−1.59)	1.23 ± 0.61 (0.89−1.36)	1.22 ± 1.01 (0.58−1.39)	2.27 ± 0.83 (1.79−2.44)	0.84 ± 0.44 (0.60−0.93)	0.82 ± 0.58 (0.35−0.81)	1.78 ± 0.90 (1.30−2.00)
RT Ankle − Flexion	1.60 ± 0.36 (1.43−1.71)	0.93 ± 0.51 (0.55−0.95)	1.01 ± 0.47 (0.67−1.03)	2.41 ± 0.51 (2.20−2.59)	0.61 ± 0.24 (0.47−0.66)	0.72 ± 0.43 (0.44−0.78)	2.19 ± 1.17 (1.67−2.58)
Pelvis − Obliquity	0.64 ± 0.18 (0.55−0.69)	0.53 ± 0.37 (0.25−0.54)	0.60 ± 0.28 (0.39−0.61)	0.76 ± 0.27 (0.67−0.87)	0.36 ± 0.21 (0.21−0.37)	0.44 ± 0.26 (0.24−0.44)	0.62 ± 0.20 (0.54−0.70)
Pelvis − Flexion	0.62 ± 0.16 (0.57−0.69)	0.70 ± 0.28 (0.54−0.76)	0.71 ± 0.51 (0.38−0.78)	1.03 ± 0.28 (0.86−1.08)	0.45 ± 0.28 (0.26−0.47)	0.45 ± 0.27 (0.28−0.48)	1.24 ± 0.62 (0.86−1.35)
Pelvis − Rotation	X	0.88 ± 0.64 (0.45−0.95)	0.92 ± 0.46 (0.64−1.00)	0.84 ± 0.37 (0.56−0.85)	0.33 ± 0.13 (0.25−0.35)	0.44 ± 0.18 (0.30−0.44)	0.53 ± 0.23 (0.42−0.60)

Mean root mean squared error (RMSE) and mean range of motion error (ROME) of the rigid marker cluster (RMC) evaluation of all subjects ± standard deviation (SD); brackets contain 95% confidence interval (CI). Columns show the results for gait, bilateral squat (SQ), single-leg squat (SLS), and countermovement jump (CMJ).

The following considers only the results of the functional movements. The RMC evaluation of the IMU data revealed RMSE and ROME to be below 3° for all joints and all movements. The highest RMSE was evident in the frontal and transversal plane. The sagittal plane revealed a RMSE between 0.93° and 1.22° and a ROME between 0.60°– 1.29° for SQ and SLS. CMJ showed a higher RMSE and ROME than SQ and SLS with respect to the hip, knee, and ankle in the sagittal plane; values ranged from 1.44°– 2.48° RMSE and 1.32°– 2.19° ROME, respectively. The best outcome in the sagittal plane for all tasks was in global pelvis flexion (RMSE 0.70°– 1.03°). Concerning the joint angles in the frontal and transversal plane, SQ, SLS, and CMJ revealed similar RMSE’s. However, ankle inversion and rotation were again highest in the CMJ task. ROME in the frontal and transversal plane was also higher in the CMJ task. The global pelvis obliquity and rotation showed the best results in the frontal and transversal plane with a RMSE below 1.00° and a ROME below 0.70°.

Table 2 shows the results of the ANOVA for the inter-task comparison of the RMSE. The most significant differences were found between the CMJ task and the remaining functional movements and Gait. No significant differences were found between SQ and SLS.

**Table 2:** Results of the ANOVA.

	RMSE					
	p-value					
	Gait vs SQ	Gait vs SLS	Gait vs CMJ	SQ vs SLS	SQ vs CMJ	SLS vsCMJ
LT Hip − Abduction	**< 0.001**	x	0.124	x	**0.015**	x
LT Hip − Rotation	0.255	x	0.475	x	0.078	x
LT Hip − Flexion	0.177	x	**< 0.001**	x	**< 0.001**	x
LT Knee − Abduction	**0.007**	x	**0.009**	x	0.859	x
LT Knee − Rotation	0.376	x	0.139	x	0.790	x
LT Knee − Flexion	**< 0.001**	x	**0.002**	x	**< 0.001**	x
LT Ankle − Inversion	0.173	x	**< 0.001**	x	**0.003**	x
LT Ankle − Rotation	0.553	x	**0.001**	x	0.002	x
LT Ankle − Flexion	**0.006**	x	**< 0.001**	x	**< 0.001**	x
RT Hip − Abduction	0.104	0.305	0.170	0.515	0.658	0.796
RT Hip − Rotation	0.634	0.079	0.189	0.211	0.407	0.696
RT Hip − Flexion	0.403	0.829	**< 0.001**	0.679	**< 0.001**	**0.005**
RT Knee − Abduction	0.122	0.198	0.121	0.812	0.752	0.969
RT Knee − Rotation	0.681	0.314	**0.031**	0.599	0.187	0.498
RT Knee − Flexion	**< 0.001**	**0.002**	**0.004**	0.885	**< 0.001**	**< 0.001**
RT Ankle − Inversion	0.200	0.666	**< 0.001**	0.597	**< 0.001**	**0.003**
RT Ankle − Rotation	**0.038**	0.162	**< 0.001**	0.987	**< 0.001**	**< 0.001**
RT Ankle − Flexion	**< 0.001**	**< 0.001**	**< 0.001**	0.567	**< 0.001**	**< 0.001**
Pelvis − Obliquity	0.193	0.544	**0.043**	0.470	**0.011**	**0.030**
Pelvis − Flexion	0.228	0.386	**< 0.001**	0.900	**< 0.001**	**0.005**
Pelvis − Rotation	x	x	x	0.815	0.788	0.518

P-values of the ANOVA for inter-task comparison of the RMSE. Rows show joint angles. Each column represents the comparison between two movements. Pelvis rotation was not compared between Gait and the remaining functional movements due to drift in the global pelvis rotation of the gait data (see [18])

**Fig 3:**
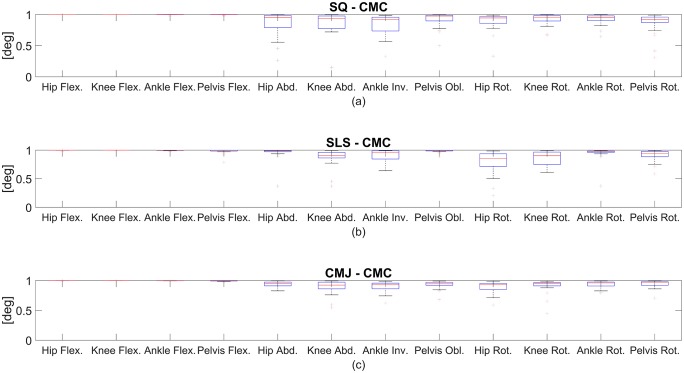
Coefficient of multiple correlation (CMC) of the RMC evaluation. CMC of the right lower extremity for all functional movements: (a) indicates SQ, (b) indicates SLS, and (c) indicates CMJ.

The CMC values were good to excellent for all joints in the SLS, SQ and CMJ tasks. The CMC values of the joint angles of the right lower extremity are plotted in Fig 3. The SQ task showed higher variances of CMC in the transversal plane as compared to SLS and CMJ tasks. SLS displayed higher uncertainties in the hip and knee rotations.

**Fig 4:**
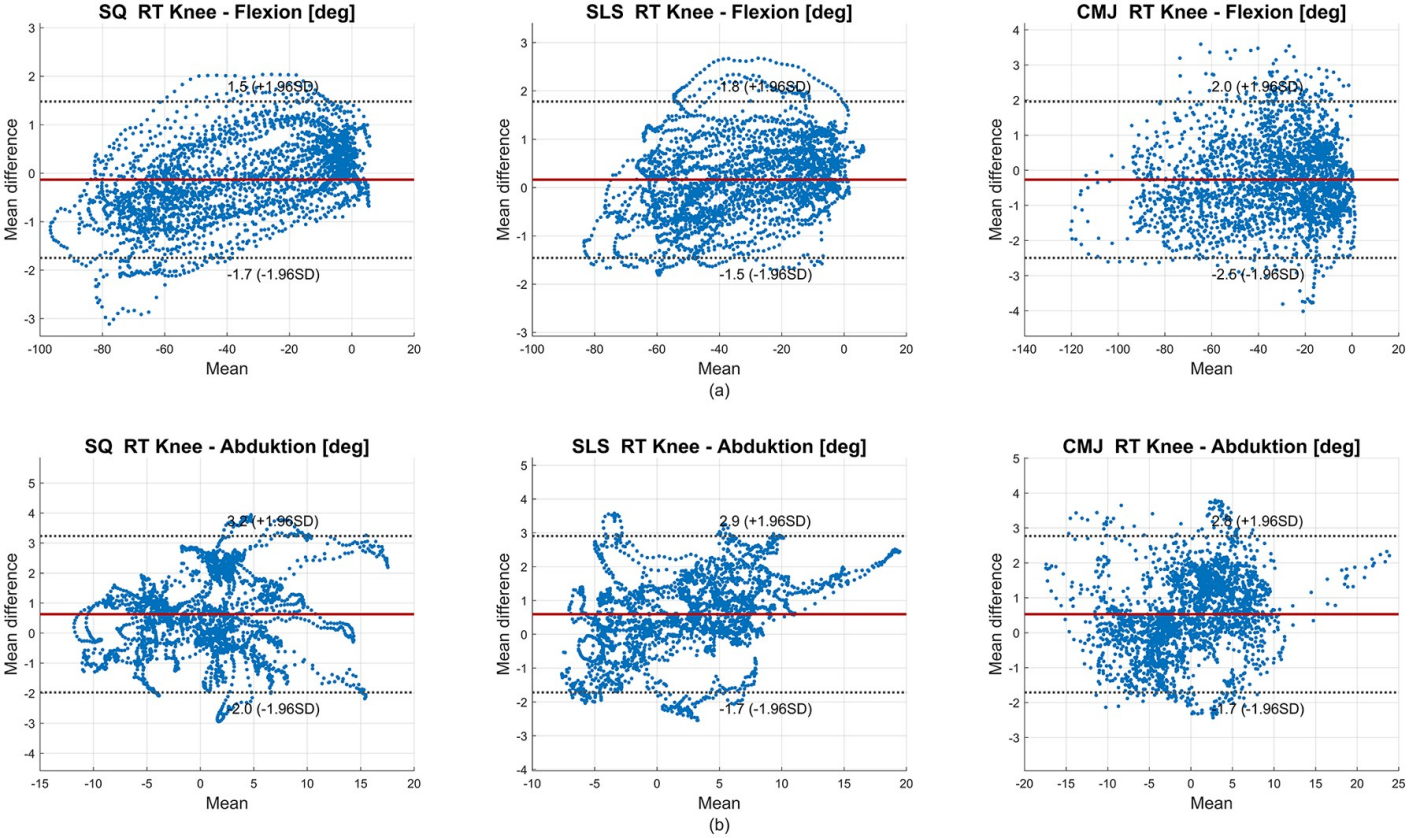
Bland-Altman (BA) diagrams of the right knee flexion (a) and abduction (b) for all subjects for the RMC evaluation. In (a), negative values on the x-axis indicate knee flexion. In (b), negative values on the x-axis indicate knee adduction. The solid line indicates the mean difference, dashed lines indicate ± 1.96 * standard deviation (SD).

A BA analysis was conducted to evaluate the limits of agreement between the IMU and OMC data in all joint angles. The SQ data revealed biases from −1.10°– 1.20° and limits ranging from ± 1.07°–± 5.06°. The SLS data displayed biases from −1.10°– 1.36° and limits ranging from ± 0.96°–± 3.25°. In the CMJ data, biases ranged from −1.34°– 1.35° and limits were between ± 1.18°–± 4.48°. Exemplary BA diagrams of the right knee flexion and abduction for all tasks are shown in Fig 4.

### 3.2 Skin marker evaluation

The RMSE and ROME of all joint angles for SQ, SLS, and CMJ are shown in Table 3. S4–S6 Figs in the supporting information show the mean joint angle waveforms of one exemplary subject in all three tasks and for all three planes.

**Table 3:** Results of the skin marker evaluation.

	RMSE [deg] ± SD (95% CI)			ROME [deg] ± SD (95% CI)		
	SQ	SLS	CMJ	SQ	SLS	CMJ
LT Hip − Abduction	3.26 ± 1.30 (2.88−3.89)	x	3.12 ± 1.56 (2.33−3.54)	3.45 ± 2.12 (2.05−3.70)	x	4.10 ± 2.76 (2.14−4.28)
LT Hip − Rotation	4.79 ± 2.40 (3.23−5.09)	x	5.26 ± 2.53 (3.86−5.82)	3.75 ± 3.15 (1.84−4.28)	x	3.12 ± 1.55 (1.98−3.18)
LT Hip − Flexion	8.27 ± 4.40 (5.42−8.84)	x	7.10 ± 3.60 (4.46−7.25)	11.08 ± 6.96 (6.66−12.05)	x	10.70 ± 6.64 (6.20−11.35)
LT Knee − Abduction	4.77 ± 2.40 (3.28−5.14)	x	4.11 ± 2.25 (2.66−4.41)	4.75 ± 3.44 (2.29−4.95)	x	3.49 ± 2.47 (2.06−3.97)
LT Knee − Rotation	4.09 ± 2.21 (2.50−4.22)	x	5.23 ± 2.36 (4.17−6.00)	3.89 ± 2.24 (2.95−4.69)	x	3.95 ± 2.08 (2.84−4.46)
LT Knee − Flexion	2.41 ± 1.82 (1.15−2.56)	x	2.84 ± 0.60 (2.59−3.05)	2.65 ± 2.39 (0.89−2.75)	x	3.43 ± 2.24 (1.84−3.58)
LT Ankle − Inversion	3.70 ± 2.07 (2.62−4.23)	x	5.00 ± 1.55 (4.31−5.51)	2.21 ± 1.58 (1.07−2.29)	x	4.47 ± 2.20 (3.51−5.22)
LT Ankle − Rotation	2.93 ± 1.82 (1.87−3.28)	x	4.27 ± 1.78 (3.46−4.84)	1.54 ± 1.00 (0.83−1.61)	x	3.04 ± 1.82 (1.62−3.03)
LT Ankle − Flexion	2.95 ± 1.42 (2.52−3.61)	x	5.32 ± 1.40 (4.80−5.89)	2.71 ± 1.68 (1.76−3.07)	x	6.23 ± 2.14 (5.54−7.20)
RT Hip − Abduction	2.69 ± 1.39 (2.14−3.22)	2.74 ± 1.66 (1.52−2.81)	2.71 ± 1.34 (1.80−2.84)	3.24 ± 2.31 (1.92−3.71)	2.05 ± 1.42 (0.84−1.94)	3.41 ± 2.39 (1.62−3.47)
RT Hip − Rotation	5.05 ± 2.77 (3.42−5.57)	5.18 ± 2.95 (3.38−5.67)	5.44 ± 3.15 (2.83−5.27)	3.83 ± 2.45 (2.93−4.83)	3.48 ± 2.06 (2.10−3.69)	3.32 ± 1.81 (2.25−3.66)
RT Hip − Flexion	7.67 ± 4.38 (5.08−8.48)	4.87 ± 3.06 (3.04−5.41)	7.11 ± 3.80 (4.79−7.74)	9.95 ± 7.47 (5.35−11.14)	5.22 ± 3.92 (2.59−5.63)	10.28 ± 6.77 (5.54−10.79)
RT Knee − Abduction	4.43 ± 2.88 (2.56−4.79)	4.77 ± 2.61 (3.08−5.11)	4.04 ± 2.18 (2.44−4.12)	5.38 ± 4.67 (1.68−5.30)	4.02 ± 3.99 (0.97−4.07)	4.45 ± 4.56 (1.60−5.13)
RT Knee − Rotation	4.08 ± 1.68 (3.05−4.35)	4.27 ± 2.64 (2.84−4.89)	5.08 ± 2.49 (3.49−5.42)	3.41 ± 2.22 (2.14−3.86)	3.42 ± 2.09 (2.38−4.00)	4.34 ± 1.86 (3.13−4.58)
RT Knee − Flexion	2.47 ± 1.33 (1.71−2.73)	2.81 ± 1.68 (1.55−2.86)	3.14 ± 1.05 (2.51−3.32)	2.86 ± 2.17 (1.63−3.31)	2.51 ± 1.74 (1.19−2.54)	3.35 ± 2.13 (1.90−3.55)
RT Ankle − Inversion	3.39 ± 1.85 (2.12−3.56)	3.58 ± 2.14 (1.92−3.58)	4.99 ± 2.01 (3.74−5.30)	2.17 ± 1.60 (1.23−2.47)	2.81 ± 1.66 (1.69−2.98)	4.32 ± 1.86 (3.21−4.65)
RT Ankle − Rotation	2.60 ± 1.45 (1.82−2.95)	3.41 ± 2.26 (1.90−3.65)	4.29 ± 2.16 (3.43−5.10)	1.56 ± 1.41 (0.31−1.40)	1.85 ± 1.11 (1.11−1.97)	3.46 ± 2.40 (1.83−3.69)
RT Ankle − Flexion	2.47 ± 1.26 (1.78−2.76)	2.81 ± 1.60 (1.61−2.85)	4.40 ± 1.42 (3.86−4.96)	2.56 ± 1.59 (2.13−3.36)	1.71 ± 1.08 (0.98−1.82)	5.33 ± 2.73 (3.49−5.61)
Pelvis − Obliquity	1.48 ± 1.02 (0.79−1.58)	1.76 ± 1.00 (1.24−2.02)	1.70 ± 0.97 (1.18−1.93)	1.22 ± 0.72 (0.70−1.26)	1.91 ± 1.44 (0.90−2.02)	1.76 ± 1.09 (1.19−2.04)
Pelvis − Flexion	4.93 ± 3.22 (2.86−5.36)	3.78 ± 2.65 (1.59−3.65)	5.35 ± 2.86 (3.42−5.64)	6.57 ± 5.11 (3.10−7.06)	3.25 ± 2.18 (1.85−3.54)	7.90 ± 5.15 (4.63−8.62)
Pelvis − Rotation	1.59 ± 1.09 (0.86−1.70)	2.12 ± 0.93 (1.75−2.48)	1.77 ± 0.97 (1.14−1.89)	0.82 ± 0.55 (0.50−0.93)	1.03 ± 0.64 (0.68−1.18)	0.95 ± 0.48 (0.66−1.03)

Mean RMSE and mean ROME of the skin marker evaluation of all subjects ± standard deviation (SD); brackets contain 95% confidence interval (CI). Columns show results for SQ, SLS, and CMJ tasks.

**Fig 5:**
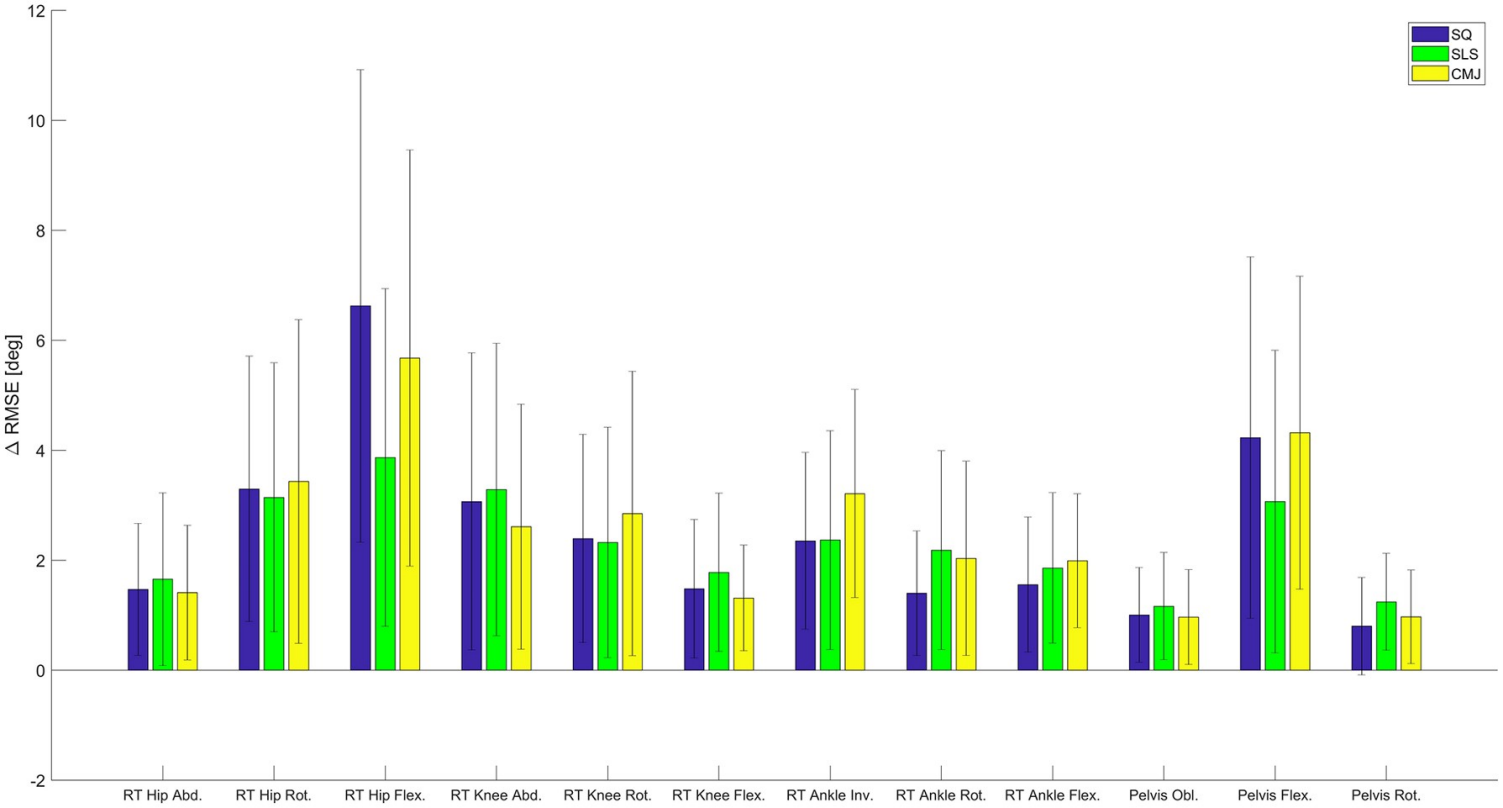
RMSE change. The mean difference in RMSE between the RMC evaluation and the skin marker evaluation in all three tasks for all joints (± SD).

**Fig 6:**
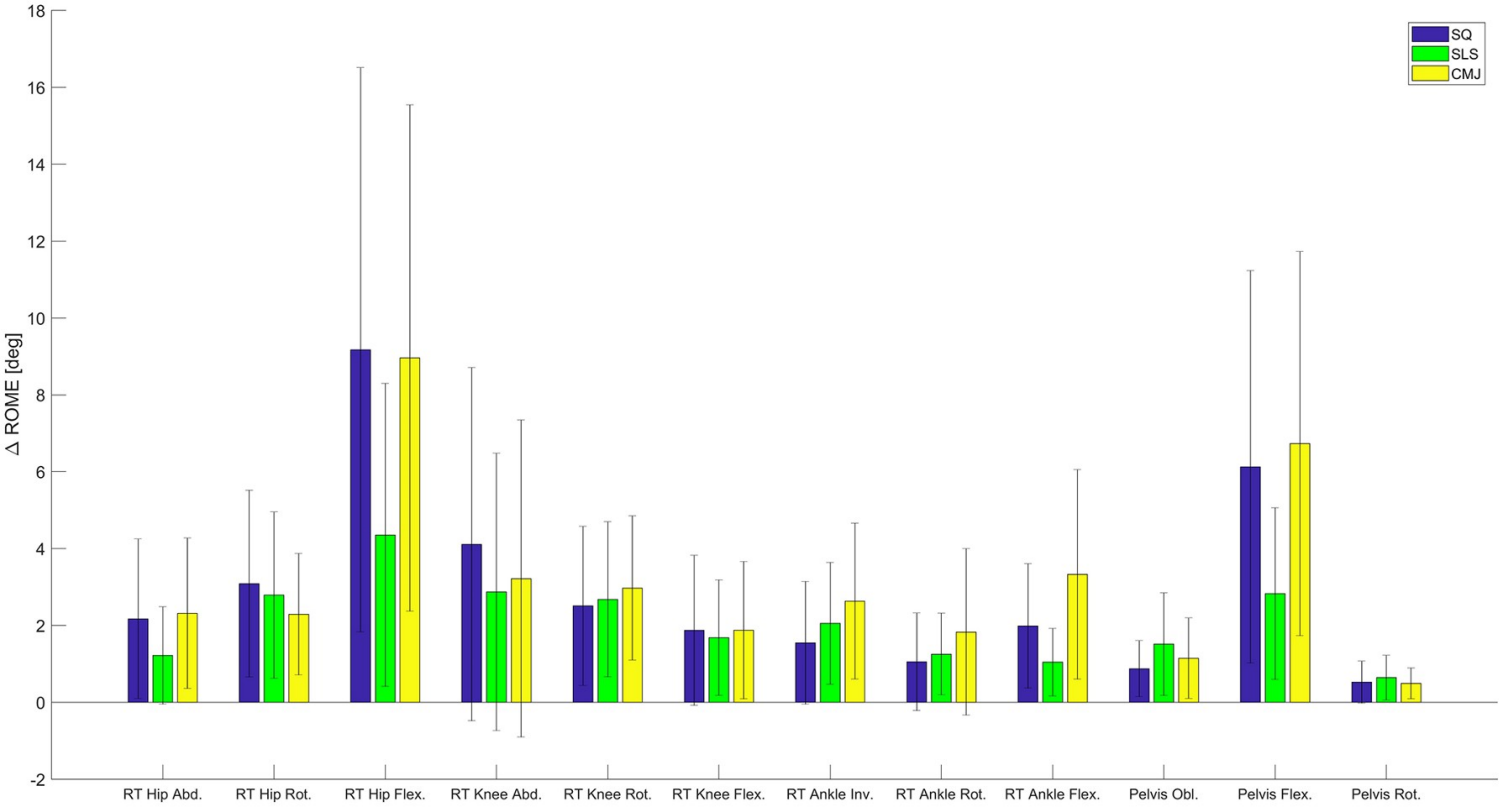
ROME change. The mean difference in ROME between the RMC evaluation and the skin marker evaluation in all three tasks for all joints (± SD).

As compared to the RMC evaluation, RMSE increased by 2.28° for SLS, 2.35° (left) and 2.40° (right) for SQ, and 2.58° (left) and 2.55° (right) for CMJ on average for all joints. The most affected joint angle was the hip joint in the sagittal plane. Hip flexion showed a RMSE of 8.27° and 7.10° in SQ and CMJ, respectively. Hip flexion in the SLS revealed a RMSE of 4.87°. The ROME was also highest for hip flexion in all three tasks with values ranging from 5.22° up to 11.08°. Figs 5 and 6 show the differences in the RMSE and ROME between the two evaluation methods for the different tasks and joint angles.

**Fig 7:**
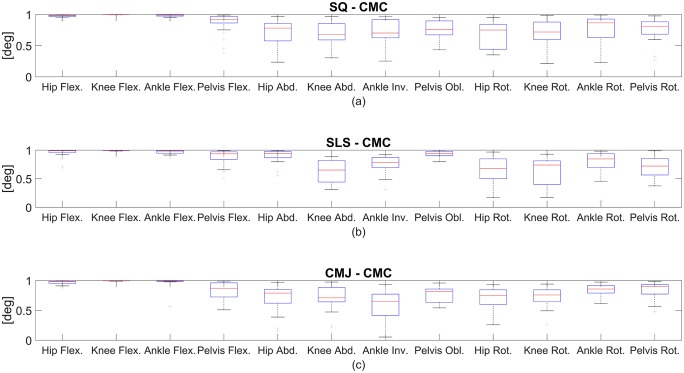
CMC in the skin marker evaluation. CMC of the right lower extremity for all functional movements: (a) indicates SQ, (b) indicates SLS, and (c) indicates CMJ.

The CMC primarily declined concerning the frontal and transversal plane. The CMC values of the joint angles of the right lower extremity are plotted in Fig 7. In the SLS task, CMC values for the mentioned planes were moderate to excellent. In the SQ and CMJ tasks, the CMC values were moderate to good. Overall, the CMC showed distinctively higher variances between the subjects in the transversal and frontal plane, which can be seen in the wide quartiles and whiskers in Fig 7.

**Fig 8:**
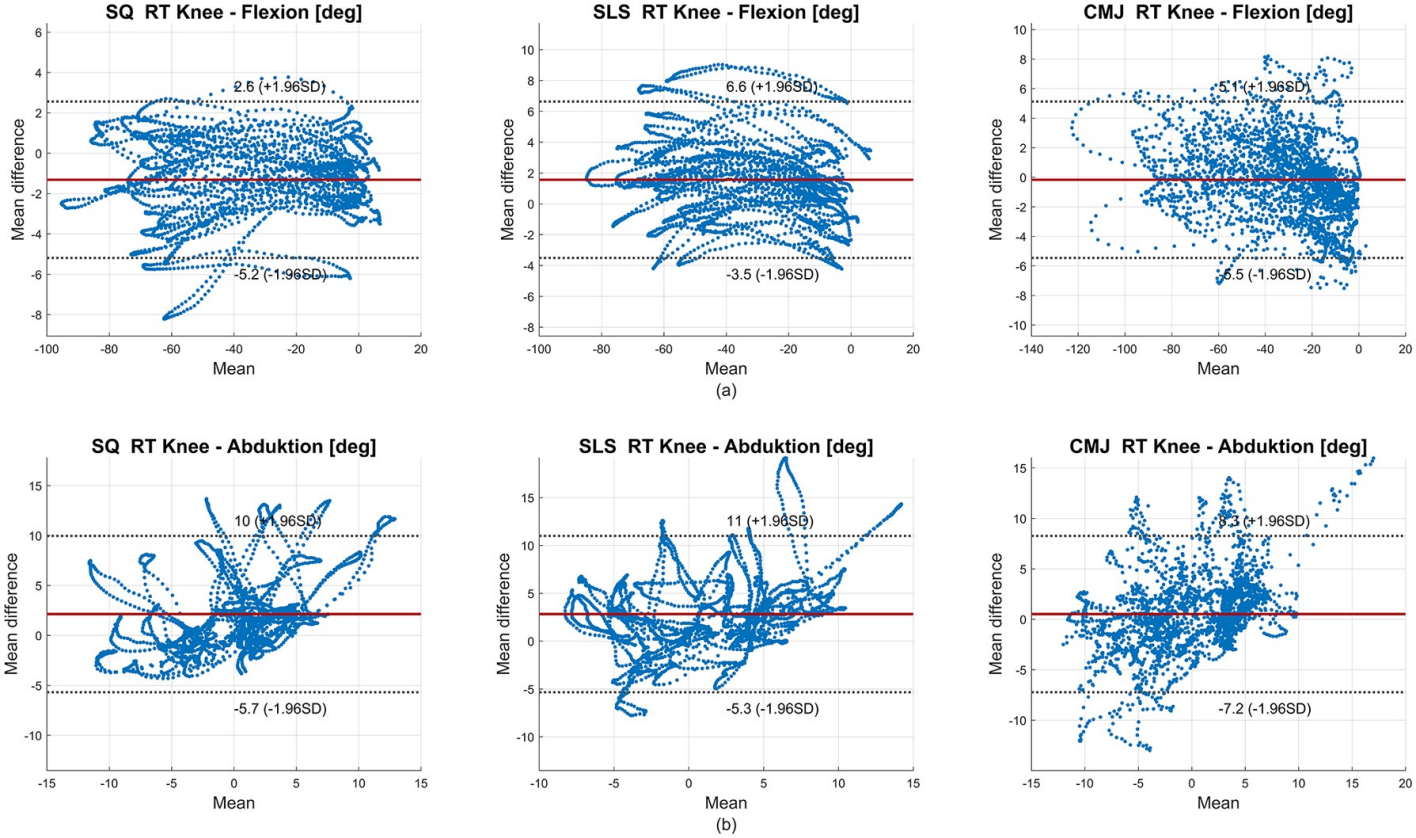
BA diagrams of the right knee flexion (a) and abduction (b) for all subjects in the skin marker evaluation. In (a), negative values on the x-axis indicate knee flexion. In (b), negative values on the x-axis indicate knee adduction. The solid line indicates the mean difference, dashed lines indicate ± 1.96 * standard deviation (SD).

The BA analysis in the skin marker evaluation revealed higher biases and limits in all tasks as compared to the RMC evaluation. SQ showed biases of −2.11°– 5.82° and limits of ± 3.09°–± 11.44°; SLS revealed biases of −1.45°– 2.89° and limits of ± 3.16°–± 9.55°; and, CMJ displayed biases of −3.54°– 4.45° and limits of ± 3.22°–± 12.47°. According to the BA analysis, the hip joint in the sagittal plane was again the most affected joint (biases 2.21°– 5.82° and limits ± 9.55°–± 12.47° for all tasks). Exemplary BA diagrams of right knee flexion and abduction in all tasks are shown in Fig 8. The movements in the remaining sagittal joint angles, as well as the global pelvis rotation, were the least affected joints.

## 4 Discussion

The primary aim of this study was to validate the accuracy of a sensor-fusion algorithm used in calculating IMU-based 3D lower extremity joint kinematics of three typical functional movements as compared to OMC joint kinematics based on RMC. The secondary aim of the analysis was to determine the error between the IMU data and the OMC data based on skin markers.

### 4.1 RMC evaluation

The RMC evaluation of 3D joint kinematics based on the IMU data of the SQ, SLS, and CMJ tasks revealed excellent correspondence with the OMC data. However, it appeared that the CMJ task revealed the highest RMSE and ROME in the sagittal plane and in ankle inversion and rotation. These findings indicate that the IMU data was influenced by the high accelerations that can occur during the landing phase in a jumping task [43]. The IMU’s used in this study employ an accelerometer with a maximum resolution of ± 16 g. A probable limitation was that the IMU recorded data with 60 Hz due to software restrictions.

The results of the ANOVA (Table 2) also revealed significantly higher RMSE in most joint angles for the CMJ (33/54) compared to the remaining tasks. The sagittal plane displayed most of the significant differences between the four tasks. That might be connected to the higher changes recorded in the ROM concerning this plane. These findings implicate that the accuracy of the measurement of joint kinematics depends on the amplitude of the movement. Significant differences were also found between Gait and SQ (7/20) as well as Gait and SLS (2/11). No significant differences were found between the SQ and SLS. However, when considering Table 1, it must be noted that the differences between tasks are in the range of the variability of the reference system itself. Thewlis et al. [44] compared two OMC systems and found differences ranging from 0.3°– 3.9° over all joint angles of the LE.

There are few studies which have examined the joint kinematics of comparable tasks using an IMU system and that excluded the uncertainties based on different magnitudes of STA using RMC [19,45]. Robert-Lachaine et al. [19] validated a commercial IMU system against an OMC system in ergonomic lifting and turning tasks, which are comparable to squatting. Their results revealed a RMSE between 1.90°– 7.30° and CMC values from 0.77–1.00. The present study showed RMSE from 0.33°– 2.92° and CMC values from 0.77–1.00 over all tasks.

In the present study, the pelvic joint kinematics was assessed as the orientation of the pelvis segment with respect to the global coordinate system. The pelvic angle’s RMSE and ROME outperformed the remaining joint angles. Lebel et al. [45] previously examined the validity of IMU orientation during a timed up-and-go test. They reported a RMSE for the global pelvis orientation for different motion sections of 1.00° (sit-to-stand), 2.20° (walk), and 1.70° (turn). The sit-to-stand movement can be viewed as a squat like motion. In this case, the present study revealed a slightly better RMSE for the global pelvic angle (0.53°– 0.88°) than had been reported.

BA analysis revealed good agreement between the OMC and IMU system. However, that the definition of acceptable limits strongly depends on the requirements of the individual application should be considered [46]. In Fig 4 knee flexion and abduction are depicted. In the SQ right knee flexion, the IMU data tended to underestimate the flexion, and conversely tended to overestimate the extension. Similar findings were revealed in the SLS right knee abduction. In this case, knee adduction tended to be underestimated, and knee abduction tended to be overestimated. However, the ROM should be unaffected by an offset, assuming that it is constant. This is important considering the value of ROM measurements in the clinical evaluation of rehabilitation progresses, for example [47]. The remaining knee joint angles seemed equally distributed within their limits.

### 4.2 Skin marker evaluation

The secondary validation of the IMU data revealed results distinctively inferior to the RMC evaluation. RMSE and ROME increased on average by approximately 2° to 3° for all tasks. The increase of the statistical error measures between the two evaluation methods was mainly associated to different effects of STA on the two systems due to the unrelated positioning of the skin markers and the IMU on the segments.

The BA analysis showed wider limits but only slightly higher mean differences. As shown in Fig 8, the data points of right knee flexion in all tasks seemed equally distributed between the limits. However, right knee abduction in all tasks tended to be overestimated by the IMU data, whereas the knee adduction tended to be underestimated.

In the skin marker evaluation, the hip flexion joint angle revealed the highest errors in the SQ and CMJ tasks. These findings are comparable to the results shown by Al-Amri et al. [14]. They found deviations of the maximum sagittal joint angle of the hip between IMU and OMC systems in the SQ and jumping tasks of approximately above 20°. However, Al-Amri et al. [14] found a static offset in their kinematic data, especially in hip flexion as well as knee and ankle rotation. An offset in the hip flexion occurred in the present study as well (S4 Fig). This might explain the rather poor results concerning the RMSE. However, CMC of the hip flexion revealed excellent correlations.

The relatively good results of the knee joint angles indicated that the offset concerning the hip sagittal joint angle did not originate from the IMU attached to the thigh. However, it appeared that the pelvis’ global sagittal angle also exhibited an offset, which directly influences the hip joint angle. However, the RMC evaluation showed no offset between the OMC and IMU data. Furthermore, the RMSE and ROME of the hip flexion in the SLS task revealed lower values compared to the SQ and CMJ tasks. Examination showed that the pelvis flexion ROM of the SLS was approximately 7.84° smaller than in the SQ task. In this case, it could have been that the skin marker based OMC system was more influenced by STA than the IMU data because markers attached to the left and right Spina Iliaca Anterior Superior were more prone to STA during increased pelvis and hip flexion. Fiorentino et al. [48] previously showed that the hip joint angle of the OMC systems based on skin markers is significantly influenced by STA, using dual fluoroscopy as a reference. They stated that the ROM of the skin marker based measurement was reduced compared to dual fluoroscopy. This is consistent with the present findings. The ROM of the hip flexion of the RMC-based OMC joint kinematics calculation was approximately 8° higher than in the skin marker based calculation, concerning the SQ task. However, the IMU based ROM calculation of the hip flexion showed similar values compared to the RMC alternative. As mentioned above, the RMC based joint kinematics calculation was shown to reveal results more immune to STA compared to joint kinematics for markers attached to anatomical landmarks [30]. Thus, the RMC calculation was considered the gold standard reference in this study. It should be considered that the susceptibility to STA of the RMC depends on their positioning on the regarding segments. However, the primary evaluation of this study focused on the performance of the sensor-fusion algorithm. In this case, accuracy should not have been sensitive to variations in the IMU placement since IMU and RMC were rigidly connected. However, future studies should investigate the effects of different IMU placement on the accuracy of 3D joint kinematics.

The RMC evaluation revealed differences between the tasks regarding the RMSE and ROME of the sagittal plane joint angle. However, in the skin marker evaluation, the hip flexion RMSE and ROME of the SQ task for the left lower extremity were higher than in the CMJ task. This could again be due to the above-mentioned susceptibility of the hip joint angle based on four markers to STA. However, differences in the error measures were around 1° between the tasks.

There are few studies that have examined the validity of IMU-derived 3D joint kinematics of dynamic tasks. Fasel et al. [31] analyzed the validity of IMU data during indoor skiing. However, their examination was performed on an indoor skiing carpet operator, thus ignoring global locomotion. They found ROME for hip and knee joint angles of 10.7° and 0.1° in the sagittal plane, 3.3° and 4.2° in the frontal plane, and 0.5° and 0.0° in the transversal plane. Thus, their results are more accurate concerning the transversal plane and the sagittal plane of the knee joint. Interestingly, the ROME regarding the hip flexion was similarly high compared to that of the present study. However, both studies differ concerning the method for joint kinematics estimation. While both studies used only gyroscope and accelerometer data for IMU joint kinematics estimation, Fasel et al. [31] also introduced a functional calibration method and initialized segment orientation using a strap down integration and joint drift reduction according to Fasel et al. [49]. In contrast, the present study obtained the calibration and segment orientation initialization of the OMC system, excluding errors regarding these issues.

## 5 Conclusion

In conclusion, the examined sensor-fusion algorithm for the calculation of IMU-based joint kinematics showed excellent correspondence with an OMC system in all three functional movements, when considering the technical error. The most dynamic task, the CMJ, showed slightly higher values for RMSE (below 3°), and ROME (below 2.5°), given a limited measurement rate of 60 Hz.

For skin markers, which are influenced by different STA compared to the IMU, the error measures increased mostly concerning the hip joint angle in the sagittal plane. However, it should be kept in mind that the reference system itself suffers from STA and uncertainties due to marker positioning. The findings mentioned above indicate that in certain cases, compared to OMC joint kinematics based on skin markers, IMU data could deliver more confident results. However, these suggestions need to be confirmed by conducting validation studies using dual fluoroscopy or comparable systems as a reference [50]. Additionally, further studies are necessary to add stepwise IMU information to the joint kinematics calculation (i.e,. an IMU-to-segment calibration, initialization from IMU data and a biomechanical model based on anthropometric tables). It is critical to separate the magnitude of error associated with each of these issues. Nevertheless, the present examination revealed results that encourage the continued research and development of an IMU system aimed at applications in rehabilitative and sports medical contexts.

## Supporting information

S1 Fig Mean sagittal plane right joint angle waveforms. Joint angle waveforms of the sagittal plane of the rigid marker cluster (RMC) evaluation of one exemplary subject. Solid lines show the joint angles of the optical motion capture system (OMC), and dashed lines show the joint angles of the inertial measurement system (IMU).
(TIF)

S2 Fig Mean frontal plane right joint angle waveforms. Joint angle waveforms of the frontal plane of the RMC evaluation of one exemplary subject. Solid lines show the joint angles of the OMC, and dashed lines show the joint angles of the IMU.
(TIF)

S3 Fig Mean transversal plane right joint angle waveforms. Joint angle waveforms of the transversal plane of the RMC evaluation of one exemplary subject. Solid lines show the joint angles of the OMC, and dashed lines show the joint angles of the IMU.
(TIF)

S4 Fig Mean sagittal plane right joint angle waveforms. Joint angle waveforms of the sagittal plane of the skin marker evaluation of one exemplary subject. Solid lines show the joint angles of the OMC, and dashed lines show the joint angles of the IMU.
(TIF)

S5 Fig Mean frontal plane right joint angle waveforms. Joint angle waveforms of the frontal plane of the skin marker evaluation of one exemplary subject. Solid lines show the joint angles of the OMC, and dashed lines show the joint angles of the IMU.
(TIF)

S6 Fig Mean transversal plane right joint angle waveforms. Joint angle waveforms of the transversal plane of the skin marker evaluation of one exemplary subject. Solid lines show the joint angles of the OMC, and dashed lines show the joint angles of the IMU.
(TIF)
