# Supplementary figures and images for: Validity of inertial sensor based 3D joint kinematics of static and dynamic sport and physiotherapy specific movements
Source: PLoS One. 2019 Feb 28;14(2):e0213064. doi: 10.1371/journal.pone.0213064 (PMC6394915; doi:10.1371/journal.pone.0213064)

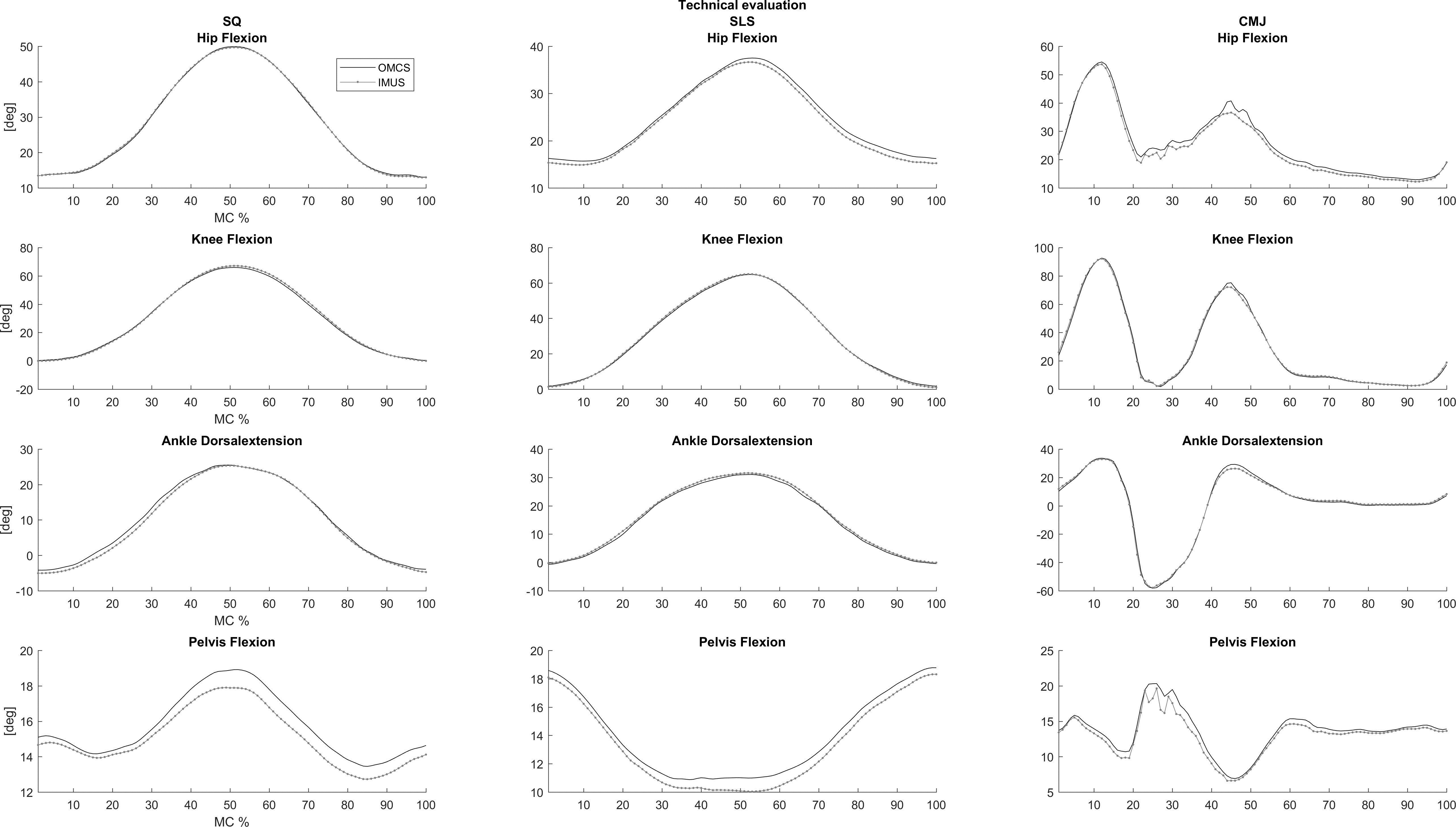

Supplement: S1 Fig — Joint angle waveforms of the sagittal plane of the rigid marker cluster (RMC) evaluation of one exemplary subject. Solid lines show the joint angles of the optical motion capture system (OMC), and dashed lines show the joint angles of the inertial measurement system (IMU). (TIF) [file pone.0213064.s001.tif]

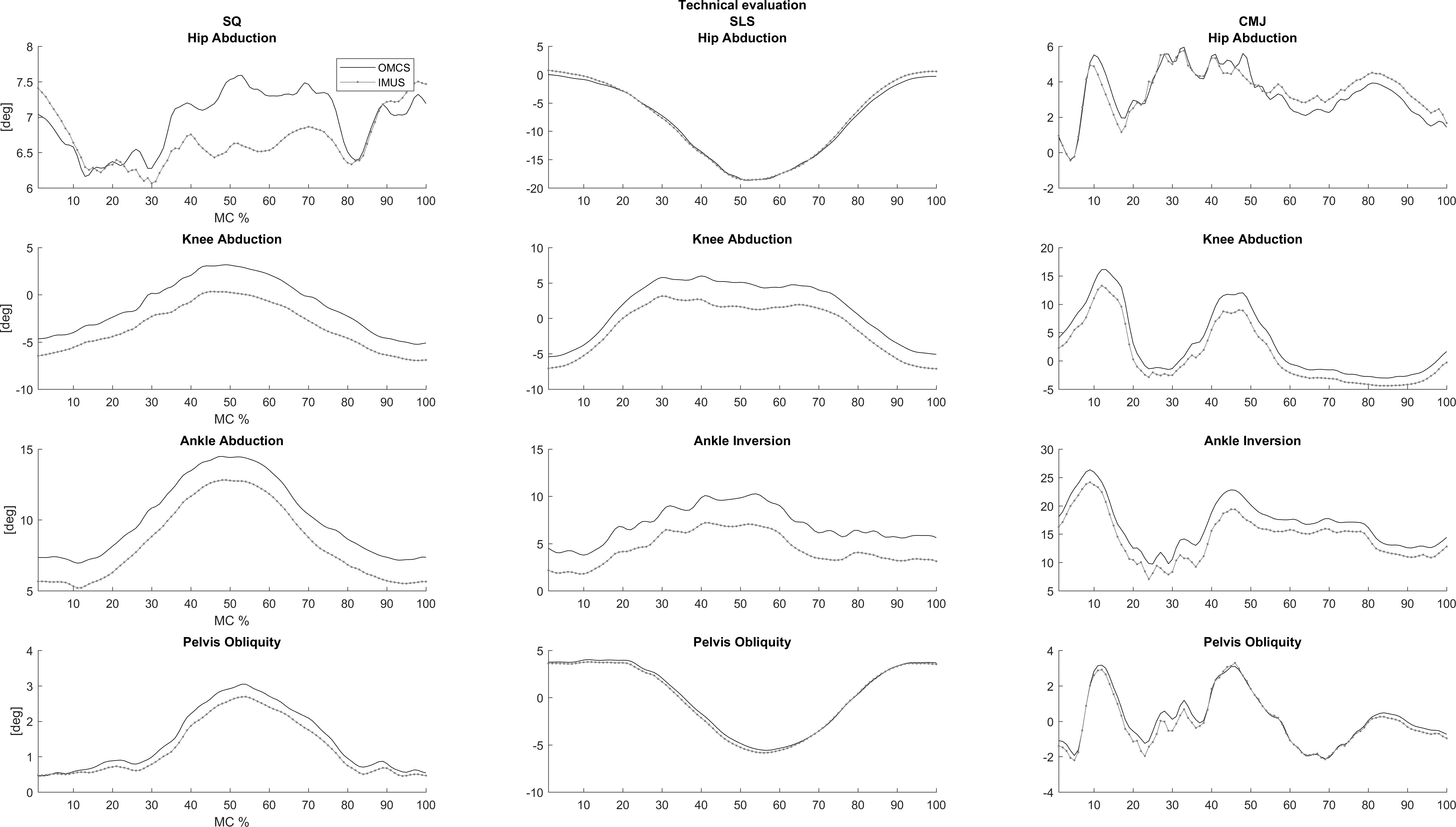

Supplement: S2 Fig — Joint angle waveforms of the frontal plane of the RMC evaluation of one exemplary subject. Solid lines show the joint angles of the OMC, and dashed lines show the joint angles of the IMU. (TIF) [file pone.0213064.s002.tif]

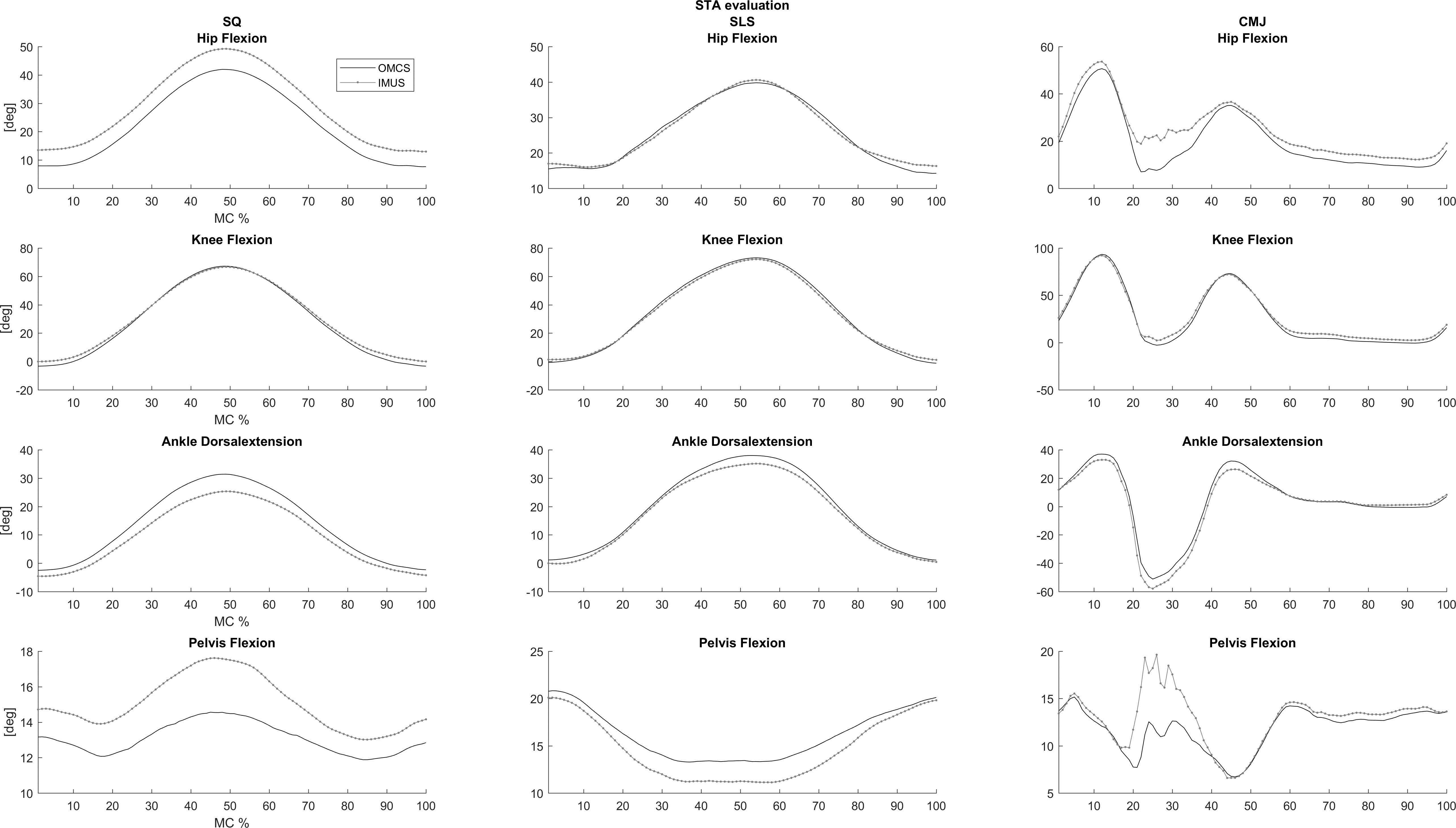

Supplement: S4 Fig — Joint angle waveforms of the sagittal plane of the skin marker evaluation of one exemplary subject. Solid lines show the joint angles of the OMC, and dashed lines show the joint angles of the IMU. (TIF) [file pone.0213064.s004.tif]

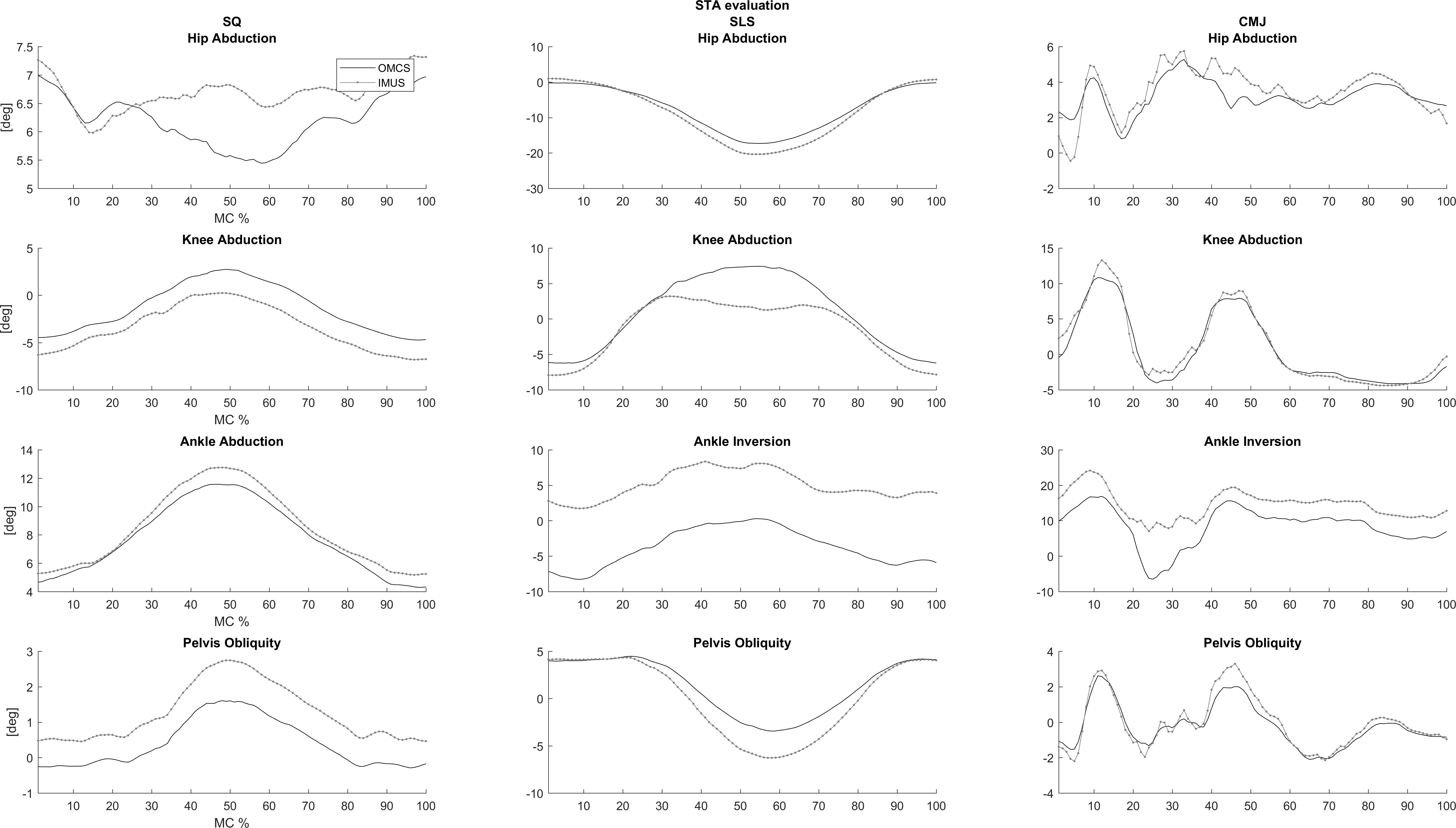

Supplement: S5 Fig — Joint angle waveforms of the frontal plane of the skin marker evaluation of one exemplary subject. Solid lines show the joint angles of the OMC, and dashed lines show the joint angles of the IMU. (TIF) [file pone.0213064.s005.tif]

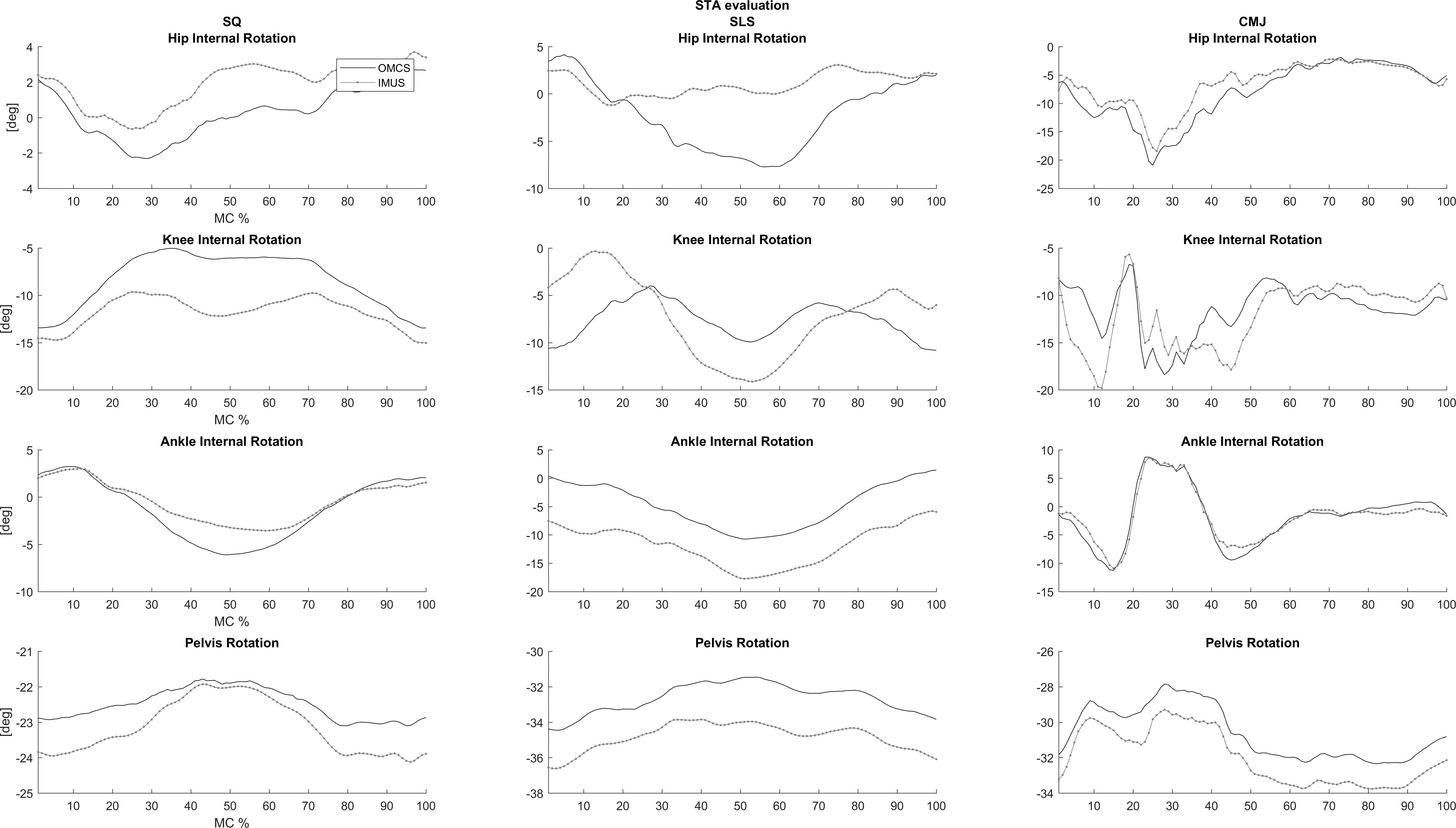

Supplement: S6 Fig — Joint angle waveforms of the transversal plane of the skin marker evaluation of one exemplary subject. Solid lines show the joint angles of the OMC, and dashed lines show the joint angles of the IMU. (TIF) [file pone.0213064.s006.tif]
